# Acyclovir Encephalopathy in HSV Encephalitis Elderly With Normal Renal Function

**DOI:** 10.1155/crnm/3854205

**Published:** 2025-10-23

**Authors:** Naoko Makishi, Yasuharu Tokuda

**Affiliations:** ^1^Department of Internal Medicine, TMG Nishi-Tokyo Chuo General Hospital, Nishitokyo, Tokyo, Japan; ^2^Department of Neurology, Musashino Emergency Hospital, Kodaira, Tokyo, Japan; ^3^Muribushi Okinawa Center for Teaching Hospitals, Urasoe, Okinawa, Japan

## Abstract

Herpes simplex virus (HSV) encephalitis is a disease treated with acyclovir (ACV). However, the neurotoxicity of ACV can lead to the development of ACV encephalopathy. While ACV is used to treat HSV encephalitis, the treatment for ACV encephalopathy is to discontinue the use of ACV. Electroencephalography (EEG) is useful for distinguishing ACV encephalopathy from HSV encephalitis. An 83-year-old male presented with fever and fatigue. He had no decline of renal function. By the fourth day, he experienced tonic-clonic seizures, right-sided conjugate deviation, and loss of consciousness, leading to a diagnosis of HSV encephalitis. He was treated with ACV, which resulted in acute kidney injury (AKI) and loss of consciousness. A diagnosis of ACV encephalopathy with AKI was confirmed by EEG. His treatment continued by vidarabine instead of ACV. His AKI improved after discontinuing ACV and performing plasma exchange, and his level of consciousness fully returned to normal. This case highlights the utility of EEG for distinguishing HSV encephalitis from ACV encephalopathy. We conducted the literature review, and AKI is considered as a risk factor for ACV encephalopathy in elderly patient using ACV or valacyclovir. However, we could not identify a cause for AKI in our case apart from the use of ACV.

## 1. Introduction

Herpes simplex virus (HSV) encephalitis, a viral encephalitis, has seen improved outcomes with the introduction of acyclovir (ACV) [1]. Also, valacyclovir (VACV), a prodrug of ACV, is used for the treatment of herpes zoster, and it can cause acute kidney injury (AKI) or ACV encephalopathy. While ACV encephalopathy is often linked to low renal function, such as in chronic kidney disease (CKD) [2], it can also occur in patients with normal renal function.

We experienced the ACV encephalopathy developed from treatment of HSV encephalitis. The patient had no CKD but developed AKI due to ACV. ACV is the first-line treatment for HSV encephalitis, and it is known for its nephrotoxicity and neurotoxicity. As the treatment for HSV encephalitis and ACV encephalopathy are exact opposite, treatment for HSV encephalitis is the use of ACV, but the treatment for ACV encephalopathy is to discontinue ACV. Therefore, we should distinguish these two diseases. Fortunately, electroencephalography (EEG) is very useful for distinguishing these two diseases, HSV encephalitis shows periodic lateralized epileptiform discharges (PLEDs) and ACV encephalopathy shows generalized slow waves. We report this case with a literature review focused on renal function evaluation.

## 2. Case Presentation

An 83-year-old man presented with fever and fatigue. His symptoms began in early July 2024, when he developed a fever and sought medical attention at our clinic, where he was diagnosed with mild heat illness and prescribed acetaminophen for symptomatic relief.

Despite the administration of the acetaminophen, fever persisted and worsened, necessitating subsequent visits to our clinic on the afternoon of the same day. During this visit, no specific source of fever was identified. He was prescribed levofloxacin along with additional acetaminophen and advised to rest. However, these medications proved ineffective, prompting another visit to the clinic at midnight. Due to the lack of improvement, the attending physician admitted him for further evaluation the following morning, referring him to the neurology department.

Upon admission, the patient's vital signs were as follows: height 171 cm, weight 66.5 kg, blood pressure 170/101 mmHg, heart rate 109 bpm, body temperature 37.7°C, and oxygen saturation 97% on room air. Neurological examination revealed no significant abnormalities, though the patient exhibited slight drowsiness. He was able to perform basic activities such as eating and walking independently, without reporting headaches.

The patient's past medical history includes Type 2 diabetes (treated with anagliptin and metformin), dyslipidemia (atorvastatin), gastroesophageal reflux disease (esomeprazole), and benign prostate hypertrophy (tamsulosin and chlormadinone). There was no notable family medical history.

The blood test results on Day 1 showed nonspecific inflammatory findings with white blood cell count of 12,740/μL, but C-reactive protein level was 0.01 mg/dL. His estimated glomerular filtration rate (eGFR) was 60 mL/min/1.73 m^2^ (Table 1). Regarding infection markers, COVID-19, Influenza A, and Influenza B antibodies were all negative.

**Figure 1:**
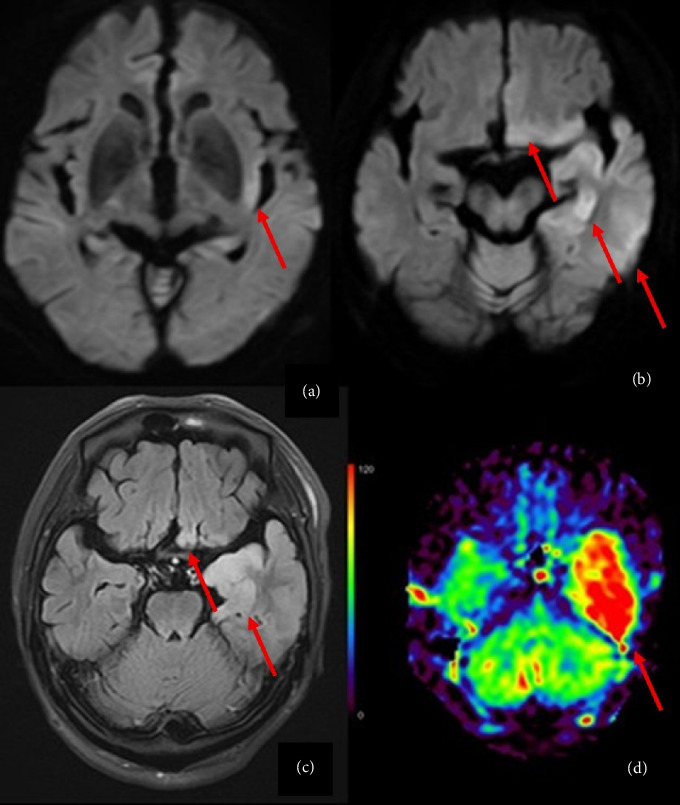
MRI on Day 1 and Day 4. (a) DWI on Day 1 showing a hyperintense region in the left temporal lobe. (b) DWI on Day 4 showing a high-signal region in the left temporal lobe, (c) FLAIR on Day 4 showing a high signal region in the left temporal lobe, and (d) ASL on Day 4 showing increased blood flow in the left temporal lobe. MRI: magnetic resonance imaging, DWI: diffusion-weighted imaging, FLAIR: fluid-attenuated inversion recovery, and ASL: arterial spin labeling.

On the evening of Day 4, the patient experienced a tonic-clonic seizure involving the entire body, accompanied by right-sided conjugate deviation and loss of consciousness. Meningitis was suspected, leading to lumbar puncture and cerebrospinal fluid (CSF) analysis, alongside magnetic resonance imaging (MRI) (Figure 1(a)). The CSF test showed a cell count of 1 μL, with a neutrophil-to-lymphocyte ratio of 1:0. The protein concentration in the CSF was 80 mg/dL, and the glucose level was 190 mg/dL (Table 1). In this time, his eGFR was 65 mL/min/1.73 m^2^. Based on these CNS findings, a diagnosis of viral encephalitis was suspected. CSF bacterial culture and HSV DNA polymerase chain reaction (PCR) tests were performed. MRI results demonstrated high signal lesions in the left temporal lobe on diffusion-weighted imaging and low signal on apparent diffusion coefficient images. Arterial spin labeling showed increased blood flow in the left temporal lobe, suggestive of herpes simplex encephalitis (Figures 1(b), 1(c), and 1(d)). Thus, HSV encephalitis was suspected. The patient was treated with ACV 625 mg every 12 h, meropenem (MEPM) 2 g every 8 h, and vancomycin (VCM) 900 mg every 12 h was initiated.

The patient developed glossoptosis and respiratory distress requiring tracheal intubation and mechanical ventilation. On Day 5, EEG revealed PLEDs. His eGFR in day was 45 mL/min/1.73 m^2^.

On Day 7, the PCR test for HSV DNA turned out to be positive. Thus, a diagnosis of HSV encephalitis was made.

Though we continued ACV, MEPM, and VCM, the patient's consciousness did not improve, prompting a second EEG on Day 14, which showed generalized delta waves indicative of encephalopathy rather than typical herpetic encephalitis. ACV encephalopathy was diagnosed, and antiviral therapy was switched to vidarabine. Plasma exchange was performed from Days 15–17 to expedite the removal of ACV. After plasma exchange, on Day 17, his eGFR recovered to 82 mL/min/1.73 m^2^.

(Regarding the original EEG images, the original image could not be obtained due to the local hospital rule).

These interventions resulted in improved consciousness to E2VTM6 on the Glasgow Coma Scale by Day 19. A third EEG on Day 21 showed no PLEDs or generalized delta waves.

**Figure 2:**
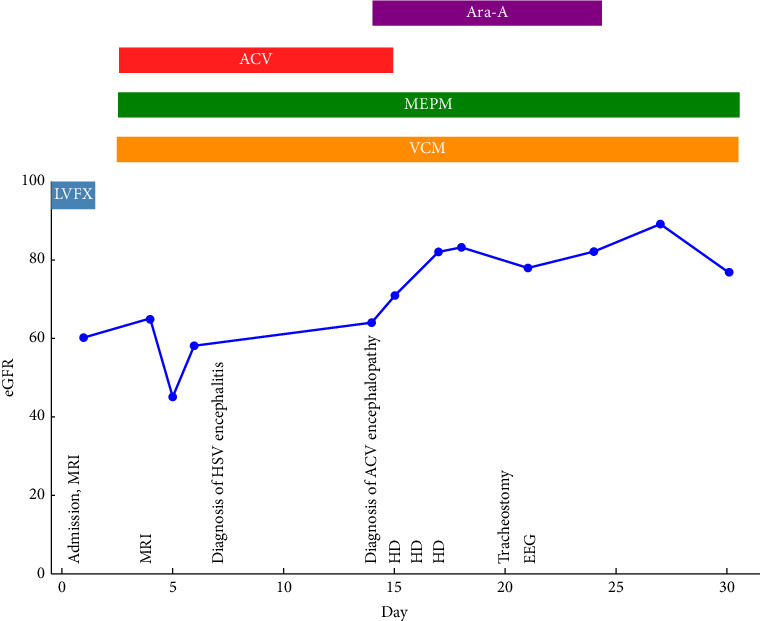
Clinical course. eGFR was significant reduced on Day 5. LVFX had been used from Day 0 to Day 1. MEPM and VCM had been used from Day 4 to Day 35 (out of chart). ACV had been used from Day 4 to Day 14. Ara-A had been used from Day 14 to Day 23. HD had been conducted from Day 15 to Day 17. Tracheostomy was constructed on Day 20. ACV: acyclovir, Ara-A: vidarabine, VCM: vancomycin, MEPM: meropenem, LVFX: levofloxacin, eGFR: estimated glomerular filtration rate, MRI: magnetic resonance imaging, PLEDs: periodic lateralized epileptiform discharges, EEG: electroencephalography, and HD: hemodialysis.

Vidarabine was discontinued on Day 22. eGFR on dDay 22 was maintained at 78 mL/min/1.73 m^2^. Although abnormal CSF results did not normalize promptly, likely influenced by prior levofloxacin use, MEPM and VCM were continued until CSF results returned to normal range by Day 35. A tracheostomy was performed on Day 20, and it was closed on Day 70. The patient was subsequently transferred to a specialized rehabilitation hospital. Description of eGFR, treatment, and events on clinical course is shown in Figure 2.

## 3. Discussion

ACV is a widely used and effective treatment for herpetic encephalitis. However, it can cause side effects such as acute renal injury (AKI) and/or ACV encephalopathy. A consciousness disorder is one of the common symptoms of both ACV encephalopathy and herpes simplex encephalitis [3]. Differentiating ACV encephalopathy from herpes simplex encephalitis is crucial, as their treatments differ. MRI (findings of inflammation including increasing blood flow in unilateral temporal lobe vs. nonspecific findings), EEG (PLEDs vs. generalized slow waves), and CSF testing (HSV DNA positive in PCR vs. nonspecify finding vs. in routine testing) are useful for distinguishing HSV/varicella zoster virus encephalitis from ACV encephalopathy [4, 5].

HSV encephalitis is due to local HSV infection and typical EEG is PLEDs, which reflects severe local inflammation. On the other hand, ACV encephalopathy is drug induced whole cerebrum encephalopathy, and EEG is generalized slow waves.

In this case, EEG was useful to distinguish ACV encephalopathy from HSV encephalitis. The characteristic EEG findings in HSV encephalitis are PLEDs, which reflect inflammation of the unilateral temporal lobes. On the other hand, EEG of ACV encephalopathy is nonspecific generalized slow waves. However, it is rare to observe generalized slow waves in HSV encephalitis.

Also, EEG is a noninvasive examination that can be performed relatively safely even in patients with impaired consciousness during the acute phase of infection.

We do not think that MEPM or VCM caused the AKI in this case because MEPM or VCM was continued even after we diagnosed the patient with ACV encephalopathy and achieved the remarkable consciousness recovery.

The risk factors for ACV encephalopathy are said to include advanced age and low renal function and obesity before ACV administration [3, 6]. However, there have been reports of patients with normal renal function prior to ACV use experiencing neurotoxicity. A previous study reported that 16.8% of ACV encephalopathy cases did not have renal failure upon admission [7].

The mechanism for AKI is suggested to involve ischemia of small arteries in the kidneys [8]. This hypothesis may explain the occurrence of AKI during ACV use in older patients. Body weight (BW) and renal function are also considered factors for AKI due to ACV use. Some studies have attempted to adjust ACV doses based on BW rather than creatinine clearance rate (CCr) in overweight patients [3].

In our literature review including the current case [8–15] (Table 2), the mean age was 79.7 ± 5.93 years. There were 5 male and 5 female cases. The mean body mass index (BMI) was 24.1 ± 6.0 kg/m^2^, without sarcopenia. Only our case used ACV as a treatment for HSV encephalitis, while others used VACV as a treatment for herpes zoster.

To convert VACV to ACV, the dosage was estimated by multiplied by a conversion factor of 0.54 [16]. We calculated the ACV dose based on ideal body weight (IBW), and IBW was calculated using body height squared and multiplied by 22. The mean dose of ACV per IBW was 11.24 ± 1.70 mg/kg, slightly exceeding the upper limit of the ACV dose per BW (10 mg/kg). There was no statistically significant difference between ACV doses per real BW or IBW (*p* value = 0.6858 in Welch's *t*-test), either per day or per single dose (*p* value = 0.1961 and 0.1868 in Welch's *t*-test) (Table 3).

Our literature review showed similar characteristics to cases of previous studies (Table 3). The eGFR in AKI cases decreased with statistical significance, albeit potentially due to publication bias. No new risk factors were identified in these studies.

The clinical factors, including age, sex, BMI, existence of hypertension and diabetes, medication (diuretics, calcium-blocker, Angiotensin II receptor blocker, angiotensin-converting enzyme inhibitor, and nonsteroidal anti-inflammatory drugs), and base renal function showed no statistical differences between Kuzume's case series [17] and our literature review. Also, in the Kuzume case series, these factors had no statistical difference between the AKI group and the non-AKI group. Thus, predictions of the occurrence of AKI in use of ACV or VACV are difficult. Thus, when we use ACV or VACV even in non-CKD patients, we should carefully check their renal function.

The small sample size of the previous case series and our review may be a limitation in identifying risk factors for ACV encephalopathy in patients with normal renal function. Therefore, a study with a larger sample size is needed.

We treat ACV encephalopathy in HSV encephalitis, paying attention to AKI due to ACV's renal toxicity. This condition concerns neurology, emergency medicine, general internal medicine, and dermatology. Awareness is crucial across these fields. Publishing case reports with detailed data such as baseline and onset renal function, height, and weight are highly desirable for accumulating insights.

## Figures and Tables

**Table 1:** Laboratory test results from blood (Day 1) and cerebrospinal fluid (Day 4), indicating mild inflammation, normal renal function, and a positive result of the HSV DNA test.

Category	Test	Result	Unit	Normal range (male)
Count of blood cells	WBC	12,740	/μL	4000–10,000
	RBC	3.96	×10^6^/μL	4.10–5.30
	Hb	13.2	g/dL	13.5–17.5
	MCV	97	fL	80–100
	PLT	185,000	/μL	150,000–350,000

Coagulation	PT-INR	0.96	—	0.9–1.1
	APTT	26.6	sec	25–35

Blood sugar	Glucose	237	mg/dL	70–109 (fasting)
	HbA1c	6.4	%	4.6–6.2

Biochemistry	Alb	4.3	g/dL	4.0–5.0
	T-bil	0.8	mg/dL	0.2–1.2
	AST	22	IU/L	10–40
	ALT	17	IU/L	5–45
	LDH	220	IU/L	120–245
	γ-GTP	25	IU/L	10–47
	CK	209	IU/L	50–200
	BUN	20.6	mg/dL	8–20
	Cre	0.92	mg/dL	0.6–1.1
	UA	3.8	mg/dL	3.6–7.0
	Na	137	mEq/L	135–145
	K	3.8	mEq/L	3.5–5.0
	Cl	101	mEq/L	98–108
	CRP	0.01	mg/dL	0.00–0.30

CSF	Cell	1	/μL	0–5
	N:L	1:0		
	Protein	80	mg/dL	15–45
	Glucose	190	mg/dL	50–80

Infection	COVID-19 Ab	Negative	—	Negative
	Flu A Ab	Negative	—	Negative
	Flu B Ab	Negative	—	Negative
	HSV DNA	Positive	—	Negative

*Note:* PLT: platelet, Alb: albumin, T-Bil: total bilirubin, AST: aspartate aminotransferase, ALT: alanine aminotransferase, Cre: creatinine, Na: sodium, K: potassium, Cl: chloride: electrolytes, N:L: neutrophil: lymphocyte ratio, WBC: white blood cell count, RBC: red blood cell count, γ-GTP = gamma-glutamyl transpeptidase.

Abbreviations: Ab = antibody, APTT = activated partial thromboplastin time, BUN = blood urea nitrogen, CK = creatinine kinase, CRP = C-reactive protein, CSF = cerebrospinal fluid, Hb = hemoglobin, HSV = herpes simplex virus, LDH = lactate dehydrogenase, MCV = mean corpuscular volume, PT-INR = prothrombin time–international normalized ratio, and UA = uric acid.

**Table 2:** Summary of case reports which show no notable features related to AKI.

Reference	Age	Sex	BH (cm)	BW (kg)	BMI	IBW	Dose as ACV (day)	Dose as ACV (a single dose)	ACV/BW (a single dose)	ACV/IBW (a single dose)	ACV dose/BW (day)	ACV dose/IBW (day)	ACV dose/BW (a single dose)	ACV dose/IBW (a single dose)
[9]	71	F	150.0	84.0	37.3	49.50	1620	540	6.43	10.91	19.29	32.73	6.43	12.52
[10]	72	F	145.0	65.3	31.1	46.26	1620	540	8.27	11.67	24.81	35.02	8.27	13.47
[15]	72	M	146.2	52.4	24.5	47.02	1620	540	10.31	11.48	30.92	34.45	10.31	11.48
[10]	78	M	168.0	60.5	21.4	62.09	1620	540	8.93	8.70	26.78	26.09	8.93	8.70
Our case	83	M	171.0	66.5	22.7	64.33	1875	937.5	14.10	14.57	28.20	29.15	14.10	14.57
[11]	83	M	163.0	50.0	18.8	58.45	1620	540	10.80	9.24	32.40	27.72	10.80	9.24
[8]	83	F	151.1	52.1	22.8	50.23	1620	540	10.36	10.75	31.09	32.25	10.36	12.33
[12]	84	F	133.8	30.2	16.9	39.39	1620	540	17.88	13.71	53.64	41.13	17.88	16.02
[13]	85	F	150.0	54.0	24.0	49.50	1620	540	10.00	10.91	30.00	32.73	10.00	12.52
[14]	86	M	153.0	51.0	21.8	51.50	1728	540	10.59	10.49	33.88	33.55	11.29	11.18

Abbreviations: BH = body height, BMI = body mass index, BW = body weight, and IBW = ideal body weight.

**Table 3:** Comparisons with previous case series, showing only eGFR has statistical difference.

	AKI group (*N* = 10) [17]	Our review (*N* = 10)	*p* value (test)
Age, years	82.70 ± 3.89	79.7 ± 5.93	0.200257 (Welch's *t*-test)
Male, *n* (%)	4 (40.0%)	5 (50.0%)	1 (Fisher's exact test)
BMI kg/m^2^	22.28 ± 2.83	24.18 ± 5.96	0.3792244 (Welch's *t*-test)
Hypertension, *n* (%)	9 (90.0%)	7 (70.0%)	0.582 (Fisher's exact test)
Diabetes, *n* (%)	2 (20.0%)	4 (40.0%)	0.6285 (Fisher's exact test)
Medication			
Diuretics, *n* (%)	1 (10.0%)	1 (10.0%)	1 (Fisher's exact test)
Ca-blocker: Calcium channel blocker	8 (80.0%)	6 (60.0%)	0.6285 (Fisher's exact test)
	4 (40.0%)	3 (30.0%)	1 (Fisher's exact test)
ARB: Angiotensin II receptor blocker	1 (10.0%)	1 (10.0%)	1 (Fisher's exact test)
	4 (40.0%)	6 (60.0%)	0.6563 (Fisher's exact test)
Before or after AKI			
Cre, mg/dL	0.86 ± 0.37	0.78 ± 0.17	0.5454496 (Welch's *t*-test)
eGFR, min/mL/1.73 m^2^	67.34 ± 37.62	65.10 ± 11.80	0.8607597 (Welch's *t*-test)
CCr, mL/min	52.32 ± 27.88	67.34 ± 20.83	0.1904695 (Welch's *t*-test)
In AKI			
Cre, mg/dL	4.31 ± 1.52	5.51 ± 1.83	0.1286679 (Welch's *t*-test)
eGFR, min/mL/1.73 m^2^	35.30 ± 10.87	13.54 ± 20.28	0.0098763^∗^ (Welch's *t*-test)
CCr, min/mL	10.03 ± 4.06	10.78 ± 6.70	0.7662942 (Welch's *t*-test)

*Note:* Ca-blocker: calcium channel blocker, Cre: creatinine.

Abbreviations: ACEI, angiotensin-converting enzyme inhibitor; AKI, acute kidney injury; ARB, Angiotensin II receptor blocker; eGFR, estimated glomerular ltration rate; and NSAIDs, nonsteroidal anti-inFammatory drugs.

^∗^ *p* < 0.001.

## Data Availability

All data generated or analyzed during this study are included in this published article.

## References

[1] Matthews E., Beckham J. D., Piquet A. L., Tyler K. L., Chauhan L., Pastula D. M. (2022). Herpesvirus-Associated Encephalitis: An Update. *Current Tropical Medicine Reports*.

[2] Lindström J., Helldén A., Lycke J., Grahn A., Studahl M. (2019). An Unexpectedly High Occurrence of Aciclovir-Induced Neuropsychiatric Symptoms in Patients Treated for Herpesvirus CNS Infection: A Prospective Observational Study. *Journal of Antimicrobial Chemotherapy*.

[3] Aboelezz A., Mahmoud S. H. (2024). Acyclovir Dosing in Herpes Encephalitis: A Scoping Review. *Journal of the American Pharmacists Association*.

[4] Kim Y. S., Jung K. H., Lee S. T. (2016). Prognostic Value of Initial Standard EEG and MRI in Patients With Herpes Simplex Encephalitis. *Journal of Clinical Neurology*.

[5] Udono M., Murayama F., Fukuda Y., Matsumoto T. (1993). Acyclovir Encephalopathy in a Herpes zoster Patient Without Renal Failure. *Japanese Journal of Clinical Dermatology*.

[6] Barber K. E., Wagner J. L., Stover K. R. (2019). Impact of Obesity on Acyclovir-Induced Nephrotoxicity. *Open Forum Infectious Diseases*.

[7] Brandariz‐Nuñez D., Correas‐Sanahuja M., Maya‐Gallego S., Martín Herranz I. (2021). Neurotoxicity Associated with Acyclovir and Valacyclovir: A Systematic Review of Cases. *Journal of Clinical Pharmacy and Therapeutics*.

[8] Hiromi S., Yuhei I., Minoru M. (2019). A Case of Acute Tubulointerstitial Nephritis After Oral Administration of Valacyclovir. *Journal of the Japanese Association of Rural Medicine*.

[9] Yamamoto A., Shindo T., Yahata S. (2024). Acyclovir Neurotoxicity due to Valacyclovir in an Elderly Patient with Normal Renal Function: A Case Report. *An Official Journal of the Japan Primary Care Association*.

[10] Nobuta H., Kitamura K., Oi E., Nishio T. (2021). Two Cases of Acyclovir–Induced Encephalopathy Treated by Hemodialysis. *Nihon Toseki Igakkai Zasshi.*.

[11] Sagawa N., Tsurutani Y., Nomura K. (2014). Acyclovir-Induced Neurotoxicity and Acute Kidney Injury in an Elderly Diabetic Patient Treated With Valacyclovir: Report of a Case. *Nippon Ronen Igakkai Zasshi. Japanese Journal of Geriatrics*.

[12] Yuya M., Akiyoshi Y., Youhei Y., Ryo Y., Midori A., Hideki M. (2021). Evaluation of Acyclovir Encephalopathy Experienced at Our Hospital. *The Journal of Japanese Red Cross Takamatsu Hospital.*.

[13] Komatsu Y., Imamura T., Kanai M. (2020). A Case of Acyclovir-Induced Encephalopathy Demonstrating an Impaired During Treatment for Herpes Zoster Which Had to Be Differentiated from Encephalitis. *Journal of Japanese Association for Acute Medicine*.

[14] Izumo A., Sakai K., Tamura Y. (2017). Acyclovir-Induced Neurotoxicity in an Elderly Patient: Report of a Case. *Journal of Japanese Society for Emergency Medicine*.

[15] Kato K., Murakami R., Shiroto H. (2022). Valacyclovir-Associated Acute Kidney Injury and Encephalopathy in an Elderly Woman with Normal Kidney Function: A Case Report. *CEN Case Report*.

[16] Acosta E. P., Fletcher C. V. (1997). Valacyclovir. *The Annals of Pharmacotherapy*.

[17] Kuzume D., Morimoto Y., Tsutsumi S., Yamasaki M., Hosomi N. (2024). Clinical Features of Acyclovir Encephalopathy Without Acute Kidney Injury. *Nippon Ronen Igakkai Zasshi Japanese Journal of Geriatrics*.

